# Gold at Cervical Border

**Published:** 1887-09

**Authors:** Wm. H. Cooke


ARTICLE VII.

### GOLD AT CERVICAL BORDER.

BY WM H. COOKE.

Much has been said of the failure of gold at the cervical margins in very large approximal cavities. The writer does not remember of having heard this complaint when soft gold was used exclusively.

Incompatibility of gold and dentine, and the shrinkage of gold under the blows of the mallet, have been assigned as causes for this failure. The writer does not accept the incompatible theory. In 1856 Mr. H—asked for treatment of upper first molar. There was extensive decay from the cervical wall to and including about one-third of grinding surface. Both horns of the pulp were exposed. The pulp was extirpated, the palatine canal, pulp chamber and cavity of decay filled with soft gold. Saw Mr. H—in 1876; found the tooth in normal condition, and no decay at cervical border. This case is one out of many, for at that day amalgam was not a popular material with which to fill teeth, being used only in teeth that were popularly termed "old shells," and in cavities that could not be made accessible. At that day the average dentist was required to fill many times more large cavities in the bicuspids and molars with gold, for the same class of patients, than at the present, and had there been failures at the cervical wall as a rule, instead of the exception, we would have heard of it.

In 1868 Mr. C—presented two superior bicuspids which had been filled with soft gold in 1844, by John Fouche, dentist, of Knoxville, Tennessee. These two teeth were widely separated with shoulders left at the gums. The cavities extended from these shoulders to near the grinding surface and were well preserved. Mr. C— requested these shoulders removed and the cavities filled with cohesive gold, and contoured in such manner as to come in contact only at the grinding surface. The shoulders were filed away and cavities shaped as deemed suitable at the cervical wall, dovetails cut in the grinding surface, and then filled with cohesive gold under the mallet, and contoured as Mr. C—wished, and to the entire satisfaction of the writer. A little more than a year afterward the patient returned for examination of those "*beautiful gold plugs.*" To the dismay of the writer the cervical and side margains had failed and the gold was only held in position by the dovetails in the grinding surface. Removing the gold with considerable difficulty, the cavities were prepared and refilled with soft gold and finished with cohesive gold as follows; after removing the decay the margins were chiseled as nearly square as possible. Abbey's soft foil No. 4 was cut into half sheets and folded into ribbons wider than the approximal depth of the cavities. These ribbons were rolled into moderately tight cylinders of sufficient size for two to cover the floor when placed in a horizontal position, the ends protruding outside the walls. These were forced in position by hand pressure and others added in like manner till the upper third of the cavities were filled. With a round, sharp-pointed instrument holes were made perpendicularly through the gold to the bottom of the cavities and fiilled with tightly rolled cyliders driven in with the mallet. This was continued as long as the instrument would penetrate the gold. The protruding ends of the cylinders were consolidated by hand pressure, trimmed and burnished before proceeding further. The middle third was filled and finished after the same manner. The upper third of both cavities were then built up with cohesive gold. cut apart with a thin file, and the gold rounded, giving the margins a good finish and leaving the gold nearly or quite touching at the grinding surface.

These teeth were examined eight years afterward, and no disintegration of tooth substance found.

These cases are referred to as illustrations why the writer concludes that soft gold properly consolidated will preserve the cervical wall as well or better than any other material, and is not incompatible with tooth substance.—*Texas Dental Journal.*

---

